# Genetic Structure and Historical Dynamics of the Economic Species *Phascolosoma esculenta* in Southeastern China

**DOI:** 10.3390/biology15060464

**Published:** 2026-03-13

**Authors:** Bohua Ma, Jiajun Zhou, Guiqing Wu, Chuan Zhu, Jiajie Zhu, Xueping Wu

**Affiliations:** Guangxi Key Laboratory of Polysaccharide Materials and Modification, School of Marine Sciences and Biotechnology, Guangxi Minzu University, Nanning 530008, China; mbh741457932@outlook.com (B.M.);

**Keywords:** *Phascolosoma esculenta*, SNP, genetic structure, historical dynamics

## Abstract

*Phascolosoma esculenta* is an economic species endemic in China and a highly prized delicacy along the country’s southeastern coast. This study focused on five *P. esculenta* populations and investigated their genetic diversity, population structure, and historical population dynamics. These populations were sampled from five locations, namely Beihai (BH) and Fangchenggang (FCG), in Guangxi; Putian, in Fujian (FJ); Danzhou, in Hainan (HN); and Zhanjiang (ZJ), in Guangdong. Genomic data were obtained through restriction site-associated DNA sequencing (RAD-seq) of 100 individuals. After quality filtering, a panel of 158,264 high-quality single nucleotide polymorphism (SNP) markers was established for subsequent analysis. The results revealed that the observed heterozygosity (*Ho* = 0.1872–0.2065) was lower than the expected heterozygosity (*He* = 0.2304–0.2382), with inbreeding coefficients (*Fis*) ranging from 0.1114 to 0.1592, indicating heterozygote deficiency and moderate inbreeding. Genetic diversity was moderate across all populations, as reflected in the values I (0.5220–0.5530), π (0.2415–0.2478), and *PIC* (0.1914–0.1982). Low genetic differentiation was observed among populations (*F*st: 0.0339–0.0509) accompanied by high gene flow (Nm = 4.6658–7.1192), suggesting ongoing genetic exchange between populations. Analysis of Molecular Variance (AMOVA) indicated that most genetic variation occurred within populations. Genetic distance and genetic similarity ranged from 0.0345 to 0.0522 and 0.9491 to 0.9661, respectively, with no significant isolation by distance (Mantel test, R = 0.0793, *p* = 0.4307). Analysis of the species’ historical population dynamics suggests that *P. esculenta* may have experienced a substantial population contraction beginning approximately 300 years ago. Overall, the five populations exhibit moderate genetic diversity, though signs of inbreeding and recent population decline may indicate early stages of germplasm degradation. These findings provide important insights for the conservation and sustainable aquaculture of this species.

## Simple Summary

*Phascolosoma esculenta* is an economic species endemic in Southeastern China, with its wild populations thought to be under threat. This study investigated its genetic diversity and population structure across five locations to inform conservation and sustainable development efforts. Results suggest all examined populations have moderate genetic diversity, with possible ongoing genetic exchange among them. Historical demographic dynamics imply a potential rapid population decline starting around 300 years ago. These findings indicate *P. esculenta* may be undergoing genetic degradation, and this research provides valuable baseline information for the conservation of wild populations and the sustainable development of its aquaculture industry.

## 1. Introduction

*Phascolosoma esculenta* (Sipuncula, Phascolosomatidae) is an economically important endemic sipunculid species in China, which is widely distributed in intertidal zones and shallow coastal waters across the southeastern coast of the country [1,2]. As a typical filter feeder and sensitive environmental pollution indicator, this species plays a vital role in maintaining the structural stability and ecological health of mangrove ecosystems, while also providing important support for coastal fishery economies [3]. Commonly known as “sea worm” or “mud worm”, *P. esculenta* is famous for its delicious taste, crisp texture and rich nutritional value, and is widely used in the production of the traditional food “Tu Sun Dong” [4]. In addition, this species possesses remarkable high medicinal value, with obvious cardiovascular protective effects including antihypertensive and antithrombotic function, which has earned it the nickname “Cordyceps of the sea” [5]. In recent years, artificial breeding and cultivation techniques of *P. esculenta* have been successfully established and achieved considerable economic benefits [6,7]. However, the seedling production still relies heavily on wild broodstocks collected from natural habitats. With the continuous expansion of market demand and the gradual deterioration of coastal marine environment, the wild germplasm resources, and genetic diversity of this species have shown a significantly downward trend, which has severely restricted the sustainable and healthy development of its aquaculture industry [8].

Genetic diversity is a fundamental component of the biodiversity which forms the core indicator to evaluate germplasm resources. At present, studies focusing on the genetic diversity and population structure of *P. esculenta* are still relatively limited. Previous studies have mainly used mitochondrial genes such as COI, cytb and D-loop, as well as a small number of microsatellite markers to carry out preliminary population genetic analyses [8,9,10,11]. Although these studies have provided basic data for understanding the genetic background of *P. esculenta*, systematic and comprehensive genome-level research is still lacking, which cannot fully meet the needs of germplasm conservation and sustainable utilization of this species.

Single Nucleotide Polymorphism (SNP) markers have become one of the most popular molecular tools in population genetics research, owing to their wide genomic distribution, high abundance good stability and efficient detection [12]. Restriction-site Associated DNA Sequencing (RAD-seq) is a high-throughput and cost-effective genotyping technology to develop molecular markers based on next-generation sequencing (NGS), which can efficiently identify and develop a large number of genome-wide SNP markers without a reference genome [13]. To date, this technology has been successfully applied in the analysis of genetic diversity and population structure in a variety of aquatic animal species, such as *Mytilus galloprovincialis* [14], *Oreochromis niloticus* [15], *Lota lota* [16], *Micropterus floridanus* [17], *Creteuchiloglanis macropterus* [18], and *Acipenser sinensis* [19]. However, the genetic diversity of *P. esculenta* has not yet been evaluated using high-throughput sequencing-based SNP markers. Accordingly, the application of such genomic approaches would be highly valuable for achieving a more comprehensive understanding of its population structure, which could further support the formulation of effective conservation strategies.

This study aimed to apply RAD-seq technology to develop SNP markers for characterizing the genetic diversity and population structure of five geographic populations of *P. esculenta* collected from the southeast coastal regions of China. The findings obtained herein are expected to provide a reliable scientific basis for the effective conservation, rational utilization, and standardized genetic management of the wild germplasm resources of this species. Furthermore, this work may also serve as a valuable reference for future population genetic studies on other marine invertebrate species.

## 2. Materials and Methods

### 2.1. Sample Collection and Processing

A total of 100 wild individuals of *P. esculenta* were randomly collected from five geographic localities along the southern coast of China. Specimens were collected from Beihai (BH) and Fangchenggang (FCG) in Guangxi Province, with 20 individuals from each site. A total of 20 individuals were collected from Zhanjiang (ZJ) in Guangdong Province. Similarly, 20 individuals were sampled from Danzhou (HN) in Hainan Province. An additional 20 individuals were obtained from Putian (FJ) in Fujian Province (Table 1). Pairwise linear distances between sampling sites ranged from 172 km to 1274 km as presented in Table 2. Only healthy wild adults with active motility, no visible external injuries and uniform body size were utilized in this study. The average body weight of these individuals was 20.00 ± 0.13 g. All specimens were transported to the laboratory under live conditions. Body wall muscle tissues were then dissected and immediately preserved in 95% ethanol. All tissue samples were stored at −80 °C until subsequent DNA extraction.

### 2.2. Genomic DNA Extraction and Quality Control

Genomic DNA was extracted from 30–50 mg of the muscle tissue using the E.Z.N.A.^®^ Mollusc DNA Kit (Omega Bio-tek, Norcross, GA, USA). DNA quality was assessed via 1% agarose gel electrophoresis and quantified using a Qubit 4.0 Fluorometer (Thermo Fisher Scientific, Waltham, MA, USA), ensuring a final concentration above 20 ng/μL. Qualified DNA samples were sent to Wuhan Feisha Gene Biotechnology Co., Ltd. (Wuhan, Hubei, China). for RAD-seq library construction.

### 2.3. RAD Library Construction and Sequencing

Genomic DNA was digested with EcoRI and DpnII. The resulting fragments were ligated to unique P1 adapters carrying sample-specific barcodes and Illumina sequencing primer sequences, together with a common P2 adapter. After ligation, products were size-selected to 300–500 bp by agarose gel electrophoresis and subsequently purified. The selected fragments were then amplified by 12 PCR cycles to enrich RAD tags and incorporate complete Illumina sequencing adapters. Finally, the multiplexed libraries were sequenced in paired-end 150 bp reads on an Illumina HiSeq X Ten platform at Wuhan Frasergen Bioinformatics Co., Ltd. (Wuhan, Hubei, China).

### 2.4. SNP Discovery and Genotyping

In this study, SNP identification and genotyping were conducted using a reference-free analytical pipeline implemented in STACKS v2.68 based on RAD-seq data for *P. esculenta*. Raw sequencing reads were de-multiplexed and quality controlled using process_RAD tags to generate high-quality clean reads. The ustacks program with parameters -m 3 and -M 2 was applied to cluster reads within each individual into initial RAD tags, which were further evaluated and filtered to obtain high-confidence loci for each individual. The cstacks program with parameter -n 2 was then used to perform cross-sample clustering of loci from all individuals and construct a catalog of consensus loci. After rigorous evaluation and filtering, a final set of high-quality shared loci across all samples was generated. The populations program with parameters -r 0.50, --min_maf 0.05, --max_obs_het 0.70 and --vcf was finally used to conduct population-level genotyping and produce preliminary variant sites.

### 2.5. SNP Data Filtering

The raw SNP dataset was subjected to stringent filtering using VCFtools v0.1.17. Loci with a mean sequencing depth less than 2× (--min-meanDP 2), a missing genotype rate higher than 50% (--max-missing 0.5), and a minor allele frequency (MAF) lower than 0.05 (--maf 0.05) were excluded stepwise across all individuals. After these filtering procedures, a high-quality SNP dataset, hereafter referred to as “Dp2–miss0.5–maf0.05”, was obtained for subsequent analyses.

### 2.6. Data Analysis

Nucleotide diversity (π), expected heterozygosity (*He*), and observed heterozygosity (*Ho*) were calculated using VCFtools (v0.1.17). Inbreeding coefficients (*Fis*) and genetic differentiation (*F*st) were analyzed using Genepop (v1.0.5). A neighbor-joining (NJ) phylogenetic tree was constructed from a genetic distance matrix using TreeBest (v1.9.2). Principal coordinates analysis (PCoA) was performed using GCTA (v1.26.0). Population structure was inferred using ADMIXTURE (v1.3.0) by setting the number of ancestral clusters (K) from 2 to 6, with 10 replicates conducted for each K value. Nei’s genetic distance was calculated using the R package adegenet (v2.1.11), and Mantel tests (comparing genetic distance vs. geographical distance) were conducted using the R packages vegan (v2.6–8) and ade4 (v1.7–23). Analysis of Molecular Variance (AMOVA) was performed using the R package ade4 (v1.7-23) to partition genetic variance among hierarchical levels.

### 2.7. Historical Effective Population Size Inference

According to Liu and Fu [20], the site frequency spectrum (SFS) method does not require a reference genome, is independent of linkage between sites, and offers high resolution for inferring recent demographic changes. Based on these advantages, this study applied this method to infer the demographic history (including effective population size, Ne) of *P. esculenta* from 100 kya to 0.1 kya using Stairway Plot v2 (v2.4.1) [21]. The folded one-dimensional SFS was constructed from the filtered high-quality SNP set using easySFS [20], with the projection set to 200 haplotypes (nseq = 200) as it yielded the highest number of segregating sites. The mutation rate was set to 2.4 × 10^−9^ per site per generation (calculated via Lynch’s regression coefficient based on a genome size of 1710 Mb [22,23]), and generation time was set to one year [8]. Finally, the median Ne trajectories and 95% confidence intervals were estimated from 200 bootstrap replicates in Stairway Plot.

## 3. Results

### 3.1. SNP Discovery via RAD Sequencing

RAD-seq libraries of 100 *P. esculenta* individuals were sequenced on the Illumina platform, yielding a mean of 14,004,255 raw reads (2.06 Gb) per sample, with average Q20 (96.99%), Q30 (91.39%) and GC content (42.53%). After stringent quality filtering, 12,984,224 clean reads (1.88 Gb) were retained per sample, with elevated Q20 (98.07%) and Q30 (93.48%) and consistent GC content (42.50%) (Table S1). Raw SNP calling via the STACKS pipeline generated 3,622,965 initial SNPs, and rigorous filtering yielded a final set of 158,264 high-confidence SNPs (Table S2). Key SNP-level quality parameters were systematically evaluated to verify dataset reliability, with sequencing depth prioritized. The final dataset had a mean sequencing depth of 15.18× per locus, with 78.63% of loci at 5–15× and no SNPs at <5× (Table S3). The global mean missing rate was 38.87%, with 85.08% of SNPs at 30–50% missing rate (no SNPs at <10% or ≥50%) (Table S4). The mean observed heterozygosity was 20.22%, with the largest fraction (40.73%) at 10–20% heterozygosity (Table S5). All raw sequence data are deposited in the NCBI under BioProject accession PRJNA1190059.

### 3.2. Evaluation of Genetic Diversity in Five Populations of Phascolosoma esculenta

The results of the genetic diversity analysis (Table 3) indicated that Shannon’s information index (I) ranged from 0.5220 to 0.5530. The observed heterozygosity (*Ho*) ranged from 0.1872 to 0.2065, while the expected heterozygosity (*He*) ranged from 0.2304 to 0.2382. All five populations exhibited *Ho* values lower than their corresponding *He* values. Nucleotide diversity (π) ranged from 0.2415 (lowest) in the FJ population to 0.2478 (highest) in the ZJ population. Polymorphism information content (*PIC*) ranged between 0.1914 and 0.1982. The inbreeding coefficient (*Fis*) varied from 0.1114 to 0.1592, with the ZJ population exhibiting the lowest value and the HN population the highest value.

### 3.3. Genetic Differentiation in Five Populations of Phascolosoma esculenta

The genetic differentiation coefficient (*F*st) and gene flow (Nm) were calculated among the five populations (Table 4). The results showed that pairwise *F*st values among populations ranged from 0.0339 to 0.0509, the lowest value (0.0339) was observed between the FCG and ZJ populations, while the highest (0.0509) was found between the ZJ and FJ populations. Except for the ZJ-FJ population pair, all other pairwise *F*st values were below 0.05. For gene flow (Nm), pairwise values ranged from 4.6658 to 7.1192, the lowest (4.6658) was observed between the FCG and ZJ populations, whereas the highest (7.1192) was found between the ZJ and FJ populations. Furthermore, all Nm values exceeded 4.

Based on the results of AMOVA (Table 5), genetic variation among populations accounted for 2.91% of the total genetic variation, whereas genetic variation within populations accounted for 97.09% of the total genetic variation.

### 3.4. Genetic Structure Analysis in Five Populations of Phascolosoma esculenta

**Figure 1:**
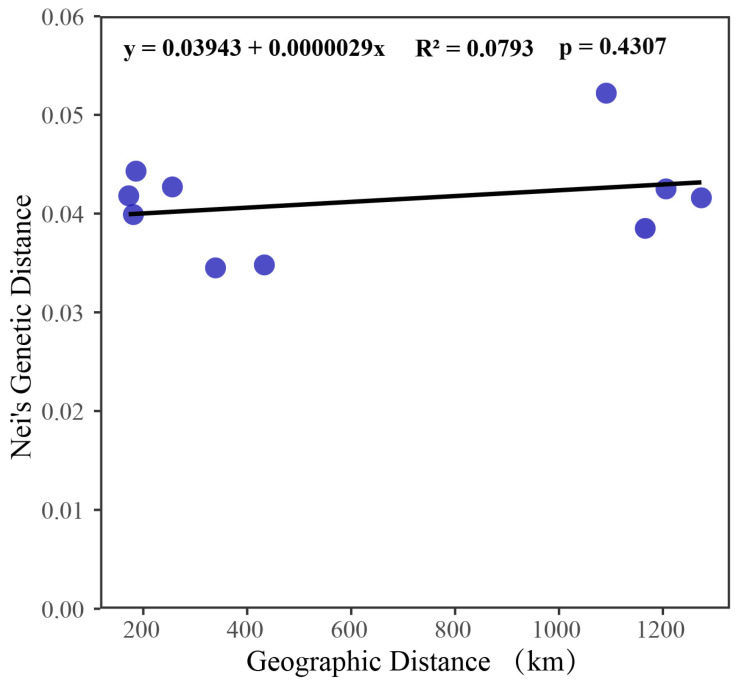
Mantel test of geographical distance and genetic distance among five *Phascolosoma esculenta* populations along the southeastern coast of China. Each point represents a pairwise comparison between populations. The X-axis represents geographic distance (km), and the Y-axis represents Nei’s genetic distance.

As shown in Table 6, the pairwise genetic distances and genetic identity among the five populations ranged from 0.0345 to 0.0522 and 0.9491 to 0.9661, respectively. The FCG and ZJ populations had the smallest genetic distance (0.0345) and the highest genetic similarity index (0.9661), whereas the ZJ and FJ populations exhibited the largest genetic distance (0.0522) and the lowest genetic similarity index (0.9491). Results of the Mantel test showed a *p*-value of 0.4307 and an R-value of 0.0793, indicating a weak correlation between geographic distance and genetic distance (Figure 1).

**Figure 2:**
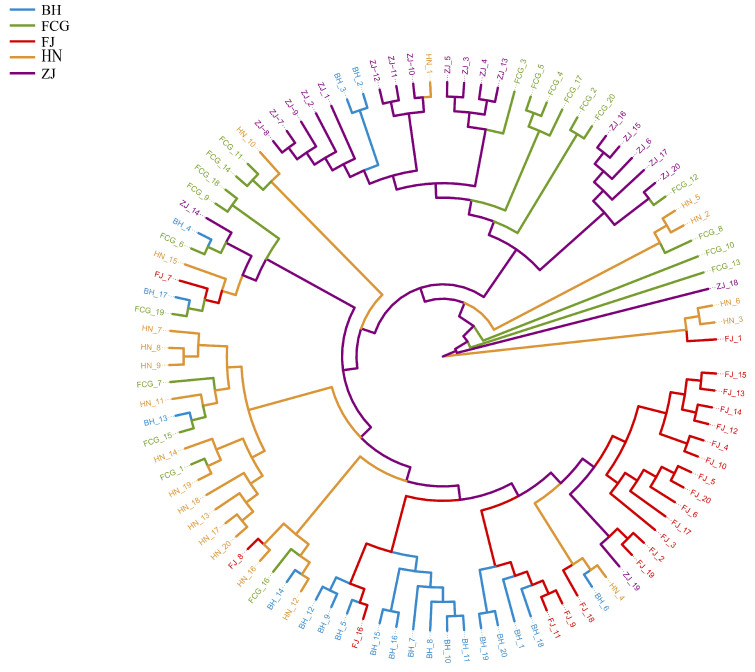
Neighbor-joining (NJ) phylogenetic tree of five *Phascolosoma esculenta* populations along the southeastern coast of China, constructed based on Nei’s genetic distances. Branch lengths represent genetic distances. Different branch colors and terminal label shapes distinguish individuals from the five geographical populations (BH, FCG, FJ, HN, ZJ). The scale bar indicates genetic distance.

The phylogenetic tree constructed using 100 *P. esculenta* samples (Figure 2) indicated that, except for the FCG population—whose samples were relatively dispersed and intermixed with those of other populations—the samples of the remaining four populations were relatively clustered within their respective populations. However, none of these clusters formed distinct monophyletic groups. Overall, sample clustering did not conform to geographic distribution patterns, as samples from different geographic populations exhibited cross-clustering.

**Figure 3:**
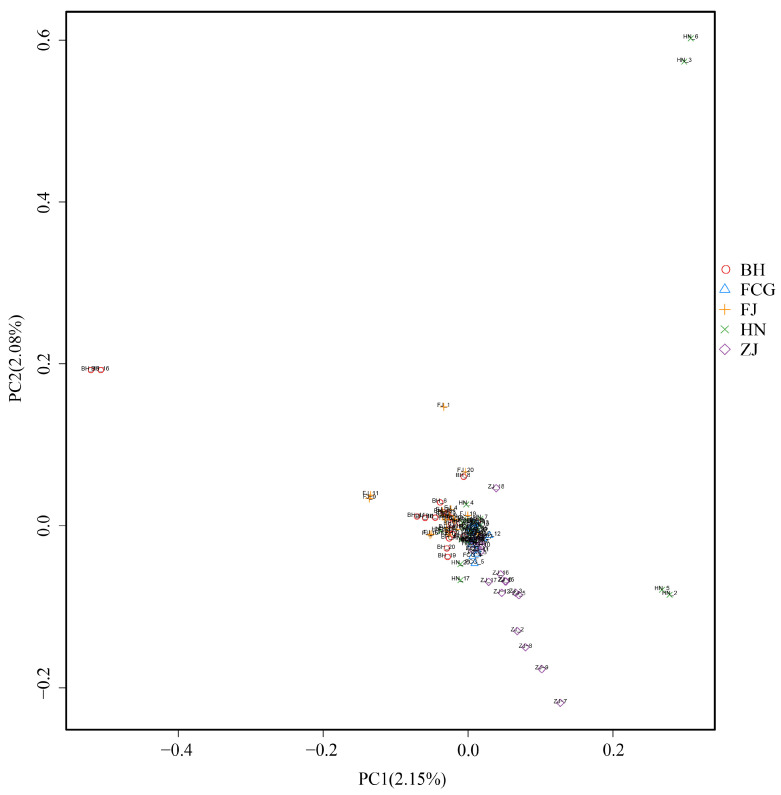
Principal Coordinate Analysis (PCoA) of five *Phascolosoma esculenta* populations along the southeastern coast of China. Scatter plot showing the distribution of all individuals along the first two principal coordinates. The X-axis represents the first principal coordinate (PC1), which explains 2.15% of the total genetic variance, while the Y-axis represents the second principal coordinate (PC2), which explains 2.08% of the total genetic variance. Dots in different colors represent individuals from distinct geographical groups (BH, FCG, FJ, HN, ZJ), illustrating genetic similarities and clustering trends among samples.

Results of principal coordinates analysis (PCoA) (Figure 3) showed that the variance explained by PC1 was 2.15%, and that by PC2 was 2.08%. From the two-dimensional PCoA plot generated by PC1 and PC2, it can be observed that among the 100 *P. esculenta* samples, most were relatively concentrated and did not form independent clusters or exhibit obvious clustering patterns. In contrast, a small number of samples from Beihai (Guangxi), Putian (Fujian), and Danzhou (Hainan) were scattered outside the main cluster, showing a discrete distribution.

**Figure 4:**
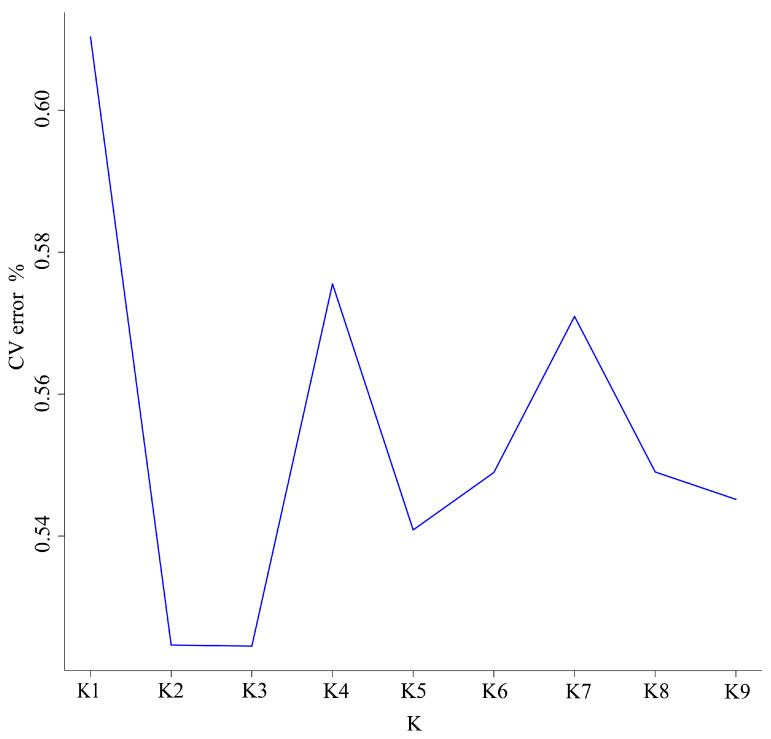
Cross-validation (CV) errors used to infer the optimal number of genetic clusters (K) for five *Phascolosoma esculenta* populations collected from the southeastern coast of China. The X-axis represents the number of genetic clusters (K), and the Y-axis represents the cross-validation error rate (CV error, %). The optimal K-value is indicated by the lowest CV error or the point where the curve begins to plateau.

**Figure 5:**
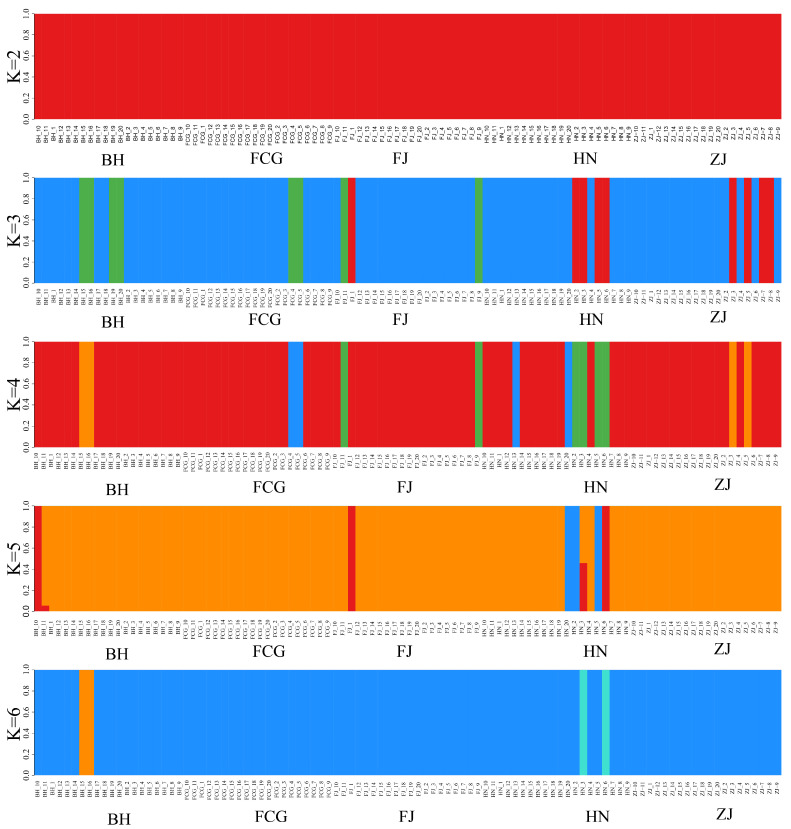
Population genetic structure of five *Phascolosoma esculenta* populations along the southeastern coast of China inferred by Bayesian clustering analysis (STRUCTURE) for K = 2–6. Each vertical bar represents a single individual, partitioned into K colored segments indicating the proportional membership (ancestry coefficient) in each genetic cluster. The X-axis denotes individual samples grouped by five sampling locations (BH, FCG, FJ, HN, ZJ). The Y-axis represents the ancestry proportion (ranging from 0 to 1).

The genetic structure of the five *P. esculenta* populations was assessed using ADMIXTURE v1.3.0. K values ranging from 1 to 9 were tested, and the resulting cross-validation (CV) error rates were examined to infer the number of genetic clusters. At K = 1, the CV error was the highest (exceeding 0.6) and thus discarded. The lowest CV errors (below 0.54) were observed at K = 2 and K = 3, however, these K values did not clearly assign the five populations to distinct subgroups, indicating no significant genetic stratification. For K = 4 and K = 7, CV errors ranged between 0.56 and 0.58, whereas at K = 5, K = 6, K = 8, and K = 9, CV errors were slightly lower (between 0.54 and 0.56), leading to the same conclusion (Figure 4). The STRUCTURE bar plot (Figure 5) also showed no distinct clusters, which corroborates the PCoA results. Overall, the results of population structure analysis were consistent with those of the PCoA, phylogenetic tree, K-value and STRUCTURE analyses.

### 3.5. Historical Dynamics in Five Populations of Phascolosoma esculenta

**Figure 6:**
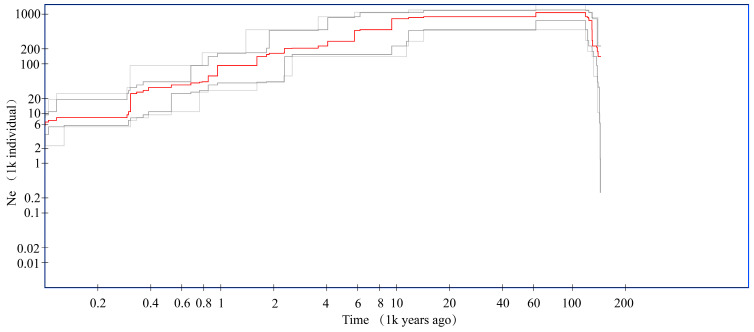
Demographic history of *Phascolosoma esculenta* along the southeastern coast of China inferred by Stairway Plot using the folded site frequency spectrum (SFS). Changes in effective population size (Ne) are plotted over the past 100,000 years. The solid red line indicates the median Ne estimate, while the grey lines delineate the 95% confidence intervals (upper and lower bounds). The X-axis represents time in thousands of years before present (kya), and the Y-axis represents effective population size in units of thousands of individuals (×10^3^ individuals).

Based on the 158,264 high-quality SNP loci identified through screening, the Stairway Plot method was used to infer changes in the effective population size (Ne) of *P. esculenta* from approximately 100,000 to 100 years before present (YBP) (Figure 6). The results indicated that in the early Holocene the population size of *P. esculenta* contracted significantly. Subsequently, during the early to mid-Holocene, the population remained relatively stable. From the middle to late Holocene, the effective population size (Ne) of *P. esculenta* declined gradually. In the recent period, the population size underwent a rapid decrease.

## 4. Discussion

### 4.1. Identification of SNPs in the Genomes in Five Populations of Phascolosoma esculenta

Molecular markers have exhibited substantial development owing to their high efficiency and sensitivity, and have been widely employed as reliable tools for assessing population genetic structure and genetic diversity in aquatic organisms [24]. Featuring cost-effectiveness and high-throughput characteristics, the combination of RAD-seq technology and SNP markers has been extensively applied in population genetic studies of diverse aquatic species, including *Sinonovacula constricta* [25], *Epinephelus striatus* [26], *Argopecten irradians concentricus* [27], and *Apostichopus japonicus* [28]. By contrast, previous population genetic investigations of *P. esculenta* have mainly relied on simple sequence repeat (SSR) markers [11], as well as mitochondrial genes such as COI [9], cytb, and D-loop [29]. To date, the application of RAD-seq and SNP markers in evaluating the population genetic structure and genetic diversity of *P. esculenta* has rarely been reported. Accordingly, this study represents an initial attempt to utilize RAD-seq combined with SNP markers to analyze the genetic structure and diversity of *P. esculenta* distributed along the southern coast of China. In total, 158,264 high-quality SNP loci were identified from 100 sequenced individuals, which were subsequently used to evaluate the genetic diversity and population structure of five geographic populations. The dataset generated in this study provides valuable genomic resources for further research on *P. esculenta,* and offers a scientific basis for the conservation and sustainable utilization of its germplasm resources.

### 4.2. Genetic Diversity in Five Populations of Phascolosoma esculenta

Genetic diversity represents an essential part of biodiversity and provides a foundation for biological evolution, environmental adaptation, and species persistence over time [30]. Higher levels of genetic diversity are generally associated with stronger adaptive capacity of populations facing environmental fluctuations [31]. A suite of indices is routinely applied in population genetic diversity analyses, including observed heterozygosity (*Ho*), expected heterozygosity (*He*), Shannon’s information index (I), inbreeding coefficient (*Fis*), and polymorphism information content (*PIC*). These parameters reflect genetic variation at distinct hierarchical levels and collectively offer comprehensive insights for interpreting population genetic structure.

Heterozygosity quantifies the frequency of heterozygous loci in a population and represents one of the most widely used metrics for assessing genetic diversity [32]. For the five geographic populations of *P. esculenta*, observed heterozygosity (*Ho*) ranged from 0.1872 to 0.2065 and expected heterozygosity (*He*) varied from 0.2304 to 0.2382. *Ho* values were consistently lower than the corresponding *He* values across all populations. This trend has been previously documented in *Apostichopus japonicus* [33] and *Pelteobagrus fulvidraco* [34]. Such heterozygote deficiency is attributed to the combined effects of inbreeding and potential technical biases. As a benthic and relatively sedentary estuarine-mangrove species, adult *P. esculenta* displays a patchy distribution pattern. These life-history characteristics may facilitate local aggregation of closely related individuals and elevate the probability of non-random mating, thereby reducing observed heterozygosity [35]. Additionally, allelic dropout may occur during RAD-seq library construction and sequencing as a result of uneven sequencing depth and mild DNA degradation. This technical artifact can misclassify heterozygous loci as homozygous and lead to the underestimation of *Ho* values [36].

Shannon’s information index (I) offers an effective means of quantifying genetic diversity within populations, with its values generally exhibiting a positive correlation with the level of genetic diversity [37,38]. Specifically, higher I values tend to indicate greater genetic diversity, whereas lower values usually reflect reduced genetic variability [39]. For the five geographically distinct *P. esculenta* populations, I values ranged from 0.5220 to 0.5530. This outcome is indicative of a moderate level of overall genetic diversity in the species. The FJ population exhibited the lowest genetic diversity among all investigated groups. This finding is likely linked to environmental pollution derived from intensive shipping and industrial activities in its natural habitat, and mortality of pollution-sensitive individuals may further reduce the genetic diversity of this population [40]. These observations align with previous investigations of *Perinereis aibuhitensis* [41,42] and *Sipunculus nudus* [43], supporting the utility of Shannon’s index for reliably characterizing intra-population genetic diversity.

In addition, the inbreeding coefficient (*Fis*) offers a reliable measure of inbreeding intensity within natural populations [44]. Positive *Fis* values indicate heterozygote deficit and evident inbreeding effects, while negative values represent the lack of inbreeding [45]. All five *P. esculenta* populations showed positive *Fis* values ranging from 0.1114 to 0.1592, supporting the heterozygote deficiency detected in our analyses. This trend may arise from moderate inbreeding, technical limitations of RAD-seq genotyping, and population degradation, and is consistent with our observed genetic diversity profiles. *P. esculenta* is a sedentary benthic species with limited dispersal capacity and is thus inherently susceptible to local inbreeding in natural habitats [46]. Intensified human activities have caused severe habitat loss and fragmentation of this species in recent decades. These human impacts further increase the likelihood of consanguineous mating and exacerbate inbreeding levels in isolated populations [47].

Nucleotide diversity (π) further provides insight into individual heterogeneity and evolutionary genetic changes within populations [48]. Values of π spanned 0.2415 to 0.2478 among the five populations, with only minor inter-population differences detected. The FJ population had the lowest π value of 0.2415 and the ZJ population had the highest value of 0.2478. These estimates appear relatively higher than those previously reported by Xu et al. [29], Gao et al. [9], Jin et al. [8], and Wang [10]. Such discrepancies may be caused by disparities in sampling locations, sample sizes, and the molecular marker systems applied in different studies.

Polymorphism information content (*PIC*) was used to consistently assess the level of genetic polymorphism across the five populations [49]. For biallelic SNP markers, the theoretical maximum *PIC* value is 0.38, a value only achieved when allele frequencies are equal in a population [50,51]. In the present study, *PIC* values ranged from 0.1914 to 0.1982. This result is approximately half of the theoretical upper limit and indicates a moderate level of genetic polymorphism in *P. esculenta*. This feature suggests the presence of genetic divergence among the five geographic populations, potentially driven by geographic isolation and habitat heterogeneity across the species’ distribution range. Notably, Song et al. [11] detected considerably higher polymorphism in aquaculture-enhanced *P. esculenta* populations from Yueqing Bay of China, with *PIC* values of 0.425, 0.409, and 0.434 in these aquaculture populations. This marked difference may be attributed to multiple factors, including differences in geographic origin, environmental conditions, sampling designs, and the number and type of molecular markers employed in different studies.

In summary, SNP marker analysis revealed a moderate level of genetic diversity across the five natural geographic populations of *P. esculenta*. The observed ranges of *He* (0.2304–0.2382), *Ho* (0.1872–0.2065) and π (0.2415–0.2478) are consistent with the inherent life-history traits of this species and comparable to those reported in other broadcast-spawning marine invertebrates with limited adult dispersal capacity, including *Mesocentrotus nudus* (*He* = 0.266–0.276, *Ho* = 0.230–0.239) [52] and *Protoreaster nodosus* (*He* = 0.289–0.294, *Ho* = 0.257–0.268) [53]. *PIC* values indicate relatively low polymorphism at individual SNP loci in *P. esculenta*. However, moderate estimates of *He*, *Ho* and π confirm that these natural populations retain sufficient genetic variation to sustain adaptive potential in response to environmental changes. These results underline the importance of implementing sustained monitoring and continuous genetic research for the conservation and sustainable management of *P. esculenta* germplasm resources.

### 4.3. Genetic Differentiation Analysis in Five Populations of Phascolosoma esculenta

The inter-population genetic differentiation index *F*st is a classic, widely applied metric in population genetics for quantifying the extent of genetic divergence among natural populations [44]. Generally, higher *F*st values correspond to greater levels of genetic differentiation between populations. Interpretive thresholds for *F*st values have been widely established in previous population genetic studies. Values below 0.05 may indicate low genetic differentiation among populations. Values ranging from 0.05 to 0.15 may suggest moderate genetic differentiation. Values ranging from 0.15 to 0.25 may reflect high genetic differentiation. Values equal to or greater than 0.25 may represent very high levels of genetic differentiation [54]. In the present study, we analyzed wild populations of *P. esculenta*. The obtained pairwise *F*st values ranged from 0.0339 to 0.0509. Most pairwise comparisons yielded *F*st values below 0.05. These results may indicate only low genetic differentiation among the corresponding populations. The only exception was the pairwise comparison between the ZJ and FJ populations, which returned an *F*st value of 0.0509. This value may suggest moderate genetic differentiation between these two populations. Similar patterns of low inter-population differentiation in *P. esculenta* have been documented in previous studies by Jin et al., Gao et al. and Song et al. [8,9,11].

Gene flow (Nm)—the transmission of genetic material from one population to another through migrating individuals—causes variations in population genetics and is negatively correlated with *F*st [55]. It is generally accepted that Nm > 1 prevents significant genetic differentiation, while Nm > 4 indicates negligible differentiation [56]. In the present study, pairwise Nm values among the five *P. esculenta* populations ranged from 4.6658 to 7.1192, all exceeding 4. This confirms the absence of significant genetic differentiation among these populations. Notably, the strongest gene flow occurred between the FCG and ZJ populations (Nm = 7.1192). In contrast, gene flow between the ZJ and FJ populations was relatively weaker (Nm = 4.6658); this may reflect a reduction dispersal efficiency of gametes or larvae. Furthermore, populations from Fangchenggang (FCG), Beihai (BH, Guangxi), and Danzhou (HN, Hainan) all lie within China’s semi-enclosed Beibu Gulf. These populations maintained high gene flow levels, with ocean currents facilitating the dispersal of their planktonic gametes and larvae.

Analysis of Molecular Variance (AMOVA) was conducted to quantify the hierarchical distribution of genetic variation among the five *P. esculenta* populations. The results showed that 97.09% of the total genetic variation originated from within populations, while only 2.91% was derived from among-population differences. This result is consistent with findings reported by Song et al. [11], Xu et al. [29] and Jin et al. [8].

The observed minimal genetic differentiation among the five populations can likely be attributed to the following factors. Under the influence of strong ocean currents and prevailing summer winds, the planktonic larvae of *P. esculenta* are readily dispersed over long distances via tidal and coastal currents, which effectively promotes gene flow among geographic populations [46,57]. As an eurythermal and euryhaline species with high environmental tolerance, the natural populations of *P. esculenta* are predominantly distributed in estuarine tidal flats with similar environmental conditions. This environmental homogeneity may reduce the selective pressure driving local adaptation-related genetic differentiation [58]. The expansion of aquaculture has involved the introduction of non-native seed stocks and the escape of cultivated individuals, which may enhance artificial genetic mixing among populations [59]. Similar patterns of weak genetic differentiation despite geographic separation have been documented in numerous other aquatic species, including *Haliotis discus hannai* [60], *Aristeus antennatus* [61], *Eriocheir sinensis* [62], and *Scylla paramamosain* [63].

### 4.4. Genetic Structure in Five Populations of Phascolosoma esculenta

Genetic distance serves as a key metric for evaluating population genetic differentiation, with its magnitude reflecting the level of genetic divergence among populations [64]. Values approaching 1 generally indicate strong or even complete reproductive isolation between populations, whereas values near 0 suggest high genetic similarity and potential genetic connectivity. In the present study, pairwise genetic distances among the five *P. esculenta* populations ranged from 0.0345 to 0.0522, and genetic similarity coefficients varied between 0.9491 and 0.9661. The Fangchenggang (FCG) and Zhanjiang (ZJ) populations exhibited the lowest genetic distance (0.0345) and highest genetic similarity (0.9661), implying a relatively close genetic relationship. In contrast, the Zhanjiang (ZJ) and Fujian (FJ) populations showed the largest genetic distance (0.0522) and lowest similarity (0.9491), suggesting a slightly higher level of differentiation between these two sites. Nevertheless, all pairwise genetic distances were far below 1, indicating overall high genetic homogeneity and weak population differentiation, which was consistent with the results of *F*st and Nm analyses. To explore the relationship between genetic differentiation and geographic distance, a Mantel test was performed to detect isolation-by-distance patterns. In Mantel analysis, the *p*-value and R-value are key indicators. Generally, a *p*-value of less than 0.05 is considered statistically significant, while an R-value closer to 1 indicates a stronger correlation [65]. The results revealed a weak and statistically non-significant correlation between Nei’s genetic distance and linear geographic distance (R = 0.0793, *p* = 0.4307), indicating that no significant isolation-by-distance signal was detected within the sampling range of this study.

To systematically resolve the population genetic structure of *P. esculenta*, we integrated phylogenetic reconstruction, Principal Coordinates Analysis (PCoA), Bayesian clustering (ADMIXTURE), and STRUCTURE analysis for multi-method verification. Results from all approaches were highly consistent and collectively indicated that no obvious genetic substructure existed across the study area. The phylogenetic tree showed that, except for some individuals from Fangchenggang admixed with other lineages, individuals from the remaining populations did not form well-supported monophyletic clades or distinct geographic clusters. In the PCoA, individuals from different geographic populations overlapped extensively in the coordinate space, only a small number of individuals from Beihai, Fujian, and Hainan were slightly scattered and failed to form independent genetic groups. Under predefined K values from 2 to 6, ADMIXTURE analysis did not identify genetic clusters corresponding to the five sampling sites, and STRUCTURE analysis also detected no significant hierarchical genetic structure or geographic isolation pattern. Taken together, these results demonstrate high genetic connectivity among *P. esculenta* populations, with no obvious geographically driven genetic differentiation.

The weak genetic structure observed in this study may be jointly driven by natural dispersal, anthropogenic activities, and environmental homogenization. Specifically, *P. esculenta* possesses a planktonic pelagosphaera larval stage lasting 16–20 days, and its reproductive season (May–September) coincides with the summer southwest monsoon [46]. During this period, the Taiwan Warm Current, South China Sea Warm Current, and Guangdong Coastal Current may form a continuous dispersal corridor connecting the South China Sea and the East China Sea, which could facilitate long-distance larval transport [66,67]. Similarly, summer circulation in the Beibu Gulf may promote larval exchange between Fangchenggang (FCG) and Zhanjiang (ZJ), potentially explaining their extremely low genetic distance [68,69]. These inferences are consistent with the non-significant isolation-by-distance pattern detected in the Mantel test and support previous findings of weak population differentiation in *P. esculenta*. In addition to natural dispersal, anthropogenic activities related to aquaculture and stock enhancement may also contribute to the observed genetic homogeneity. For instance, the genetic distance between Fangchenggang (FCG) and Zhanjiang (ZJ) was lower than that between the geographically closer Beihai (BH) and Zhanjiang (ZJ), a pattern largely inconsistent with strict isolation by distance. This discrepancy could reflect frequent translocation of seed stocks, escape of cultured individuals, and large-scale stock enhancement programs across Guangxi, Guangdong, and Hainan. Such human-mediated gene flow may effectively reduce genetic differentiation and enhance connectivity among geographically separate populations [11,29]. Besides the above factors, environmental homogenization may further promote genetic uniformity across populations. All sampling sites were located in mid-to-high intertidal estuarine habitats with relatively homogeneous environmental conditions, including sandy-mud sediments, rich organic matter, and compact substrates considered suitable for larval settlement [70]. Such homogeneous selective environments may impose convergent selection pressures, thereby tending to preserve genetic uniformity among populations [71].

Collectively, prolonged pelagic larval duration, ocean current-mediated larval dispersal, aquaculture-related anthropogenic translocation, and environmentally homogenizing selection may act synergistically to alleviate geographic isolation. These combined factors could maintain high genetic connectivity and potentially result in the five *P. esculenta* populations in this study tending to constitute a single, genetically undifferentiated unit.

### 4.5. Historical Dynamics Analysis in Five Populations of Phascolosoma esculenta

The demographic history of a species is shaped by historical climatic events and intrinsic genetic properties and provides essential evidence for evaluating its adaptive potential and long-term viability [72,73]. In this study, we applied the Stairway Plot method to reconstruct the temporal dynamics of effective population size (Ne) in *P. esculenta* over the past 100,000 years. The mutation rate used was 2.4 × 10^−9^ per site per year, estimated from theoretical regression based on genome size [22,23]. Our results delineated four distinct phases of Ne fluctuation in *P. esculenta*. The first population decline occurred in the early Holocene, a period characterized by intense climatic oscillations and rapid sea-level rise [74]. Growing evidence indicates that such extreme environmental changes drive habitat loss and fragmentation in intertidal and estuarine species, as observed in marine invertebrates including *Austrovenus stutchburyi* and *Pollicipes polymerus* [75,76]. This pattern suggests that *P. esculenta* likely retreated to discrete refugia, a common demographic strategy employed by intertidal invertebrates in response to coastal disturbances [77,78]. Subsequently, Ne stabilized during the middle Holocene, coinciding with the stabilization of global climate, reduced sea-level fluctuations, and the recovery of coastal ecosystems [79,80]. This stability was likely maintained by persistent gene flow and the expansion of effective population size [81], which is critical for sustaining genetic diversity across generations. From the mid-late Holocene to the present, Ne exhibited a continuous and gradual decline. This long-term trend is tightly associated with late Holocene climatic change and increasing human exploitation of coastal resources [82,83]. Analogous population declines under anthropogenic pressure have also been reported in *Haliotis rufescens* and *Mytilus californianus* [84]. Since the early modern era, Ne of *P. esculenta* has declined markedly, which may be partially driven by intensified anthropogenic disturbances along the southeastern coast of China [85], including overharvesting [86], coastal reclamation [87], marine pollution [88], and climate-related coastal ecosystem degradation [89]. Nevertheless, our genetic data cannot definitively distinguish whether human activities or the Holocene climatic fluctuations represent the dominant driver of the observed Ne reduction. Overall, despite inherent uncertainties in mutation rate calibration, our demographic reconstructions indicate that *P. esculenta* has undergone substantial population contraction in recent history.

## 5. Conclusions

This study revealed moderate levels of genetic diversity across the five geographical populations of *P. esculenta*, with the ZJ population exhibiting the highest diversity and the FJ population the lowest. Positive *Fis* values were observed across all populations, which may indicate a certain degree of inbreeding and heterozygote deficiency. Genetic differentiation among populations was generally low, as the majority of pairwise *F*st values were below 0.05, and only moderate differentiation was detected between the ZJ and FJ populations. The genetic variation was predominantly within populations, with minimal variation among populations. No significant genetic structure corresponding to geographical distribution was detected, and no significant isolation-by-distance pattern was identified. Analysis of the species’ historical population dynamics suggests that *P. esculenta* may have experienced a substantial population contraction beginning approximately 300 years ago. In conclusion, although the genetic diversity of the five *P. esculenta* populations remains at a moderate level, the observed population decline and signs of inbreeding may indicate some degree of germplasm resource degradation. Future efforts should be made to strengthen the genetic monitoring of wild *P. esculenta* populations. In aquaculture, seedling breeding management needs to be improved to prevent inbreeding and the escape of cultivated individuals. These measures will help preserve the genetic diversity of *P. esculenta*.

## Figures and Tables

**Table 1:** Sample information of *Phascolosoma esculenta* from the southeastern coast of China.

Code	Location	Latitude (N)	Longitude (E)	Sample Size	Date
HN	Danzhou, Hainan	19.52° N	109.58° E	20	15 July 2022
BH	Beihai, Guangxi	21.70° N	109.69° E	20	8 January 2022
FCG	Fangchenggang, Guangxi	21.55° N	107.97° E	20	16 August 2022
ZJ	Zhanjiang, Guangdong	20.98° N	110.51° E	20	9 February 2022
FJ	Putian, Fujian	25.42° N	118.99° E	20	17 September 2022

**Table 2:** Pairwise geographical distance (km) among five sampling sites of *Phascolosoma esculenta* along the southeastern coast of China.

	BH	FCG	FJ	HN	ZJ
BH	-	181	1166	256	186
FCG		-	1206	433	339
FJ			-	1274	1091
HN				-	172
ZJ					-

Note: The dash (-) on the diagonal indicates that the distance between the same site is not applicable.

**Table 3:** Genetic diversity parameters in five populations of *Phascolosoma esculenta* along the southeastern coast of China.

Group	*Ho*	*He*	I	*Fis*	π	*PIC*
BH	0.1872	0.2322	0.5347	0.1583	0.2423	0.1931
FCG	0.1921	0.2353	0.5476	0.1521	0.2449	0.1960
FJ	0.1955	0.2304	0.522	0.1238	0.2415	0.1914
HN	0.1926	0.2382	0.553	0.1592	0.2477	0.1982
ZJ	0.2065	0.2378	0.546	0.1114	0.2478	0.1977

Note: *Ho* indicated observed heterozygosity, *He* indicated expected heterozygosity, I indicated Shannon’s information index, *Fis* indicated the inbreeding coefficient, π indicated the Nucleotide diversity, *PIC* indicated polymorphism information content.

**Table 4:** Genetic differentiation and gene flow among five *Phascolosoma esculenta* populations along the southeastern coast of China.

Populations	BH	FCG	FJ	HN	ZJ
BH	—	6.1478	6.3612	5.7335	5.5226
FCG	0.0391	—	5.7545	7.0591	7.1192
FJ	0.0378	0.0416	—	5.8918	4.6658
HN	0.0418	0.0342	0.0407	—	5.8519
ZJ	0.0433	0.0339	0.0509	0.0410	—

Note: Diagonal values represent within-population comparisons and are denoted by “—” as they are not applicable.

**Table 5:** AMOVA results for five *Phascolosoma esculenta* populations along the southeastern coast of China.

Source of Variation	df	Sum of Squares	Variance of Component	Percentage Variation
Among populations	4	0.148	0.000693 Va	2.91% ***
Within populations	95	2.196	0.023114 Vb	97.09% ***
Total variation	99	2.344	0.023807	100%

Note: Va represents genetic variation among populations. Vb represents genetic variation within populations. *** indicates 9999 simulation tests showed extremely significant (*p* < 0.001).

**Table 6:** Nei’s genetic distance and genetic identity among five *Phascolosoma esculenta* populations along the southeastern coast of China.

Populations	BH	FCG	FJ	HN	ZJ
BH	—	0.9609	0.9622	0.9582	0.9567
FCG	0.0399	—	0.9584	0.9658	0.9661
FJ	0.0385	0.0425	—	0.9593	0.9491
HN	0.0427	0.0348	0.0416	—	0.9590
ZJ	0.0443	0.0345	0.0522	0.0418	—

Note: Diagonal values represent within-population comparisons and are denoted by “—” as they are not applicable.

## Data Availability

The data and references presented in this study are available from the corresponding author upon reasonable request. The raw sequence data reported in this study have been deposited in the NCBI BioProject database under accession number PRJNA1190059 and are accessible via the following link: https://www.ncbi.nlm.nih.gov/bioproject/1190059 (accessed on 24 November 2024).

## Supplementary Materials

The following supporting information can be downloaded at: https://www.mdpi.com/article/10.3390/biology15060464/s1, Table S1: Sequencing and quality statistics for each of the 100 *P. esculenta* samples used in this study. Table S2: RAD-seq sequencing data and SNP sites statistics. Table S3: Distribution of SNP sites by sequencing depth range. Table S4: Distribution of SNP sites by missing data rate. Table S5: Distribution of SNP sites across heterozygosity ranges.
