# Supplementary material for: Genetic Structure and Historical Dynamics of the Economic Species Phascolosoma esculenta in Southeastern China
Source: Biology (Basel). 2026 Mar 13;15(6):464. doi: 10.3390/biology15060464 (PMC13023452; doi:10.3390/biology15060464)
Supplement: Supplementary file 1 [file biology-15-00464-s001.zip › biology-4123238-supplementary.pdf]

Table S1 Sequencing and quality statistics for each of the 100 *P. esculenta* samples used in this study.

Sample	Raw Reads	Raw Bases	Q20%	Q30%	GC%	Clean Reads	Clean Bases	Q20%	Q30%	GC%
HN1	17195878	2510598188	97.18	91.74	40.86	16120056	2332175508	98.18	93.68	40.84
HN2	5671160	833660520	96.92	91.02	41.68	5273968	767219203	98.02	93.15	41.66
HN3	5587252	821326044	96.54	89.83	42.34	5180860	753029345	97.70	92.04	42.30
HN4	5553902	816423594	96.40	89.36	48.51	5180564	750258339	97.45	91.35	48.42
HN5	21402760	3146205720	96.89	90.90	41.12	19918944	2898498602	97.99	93.04	41.11
HN6	19534046	2871504762	96.48	89.64	42.29	18104170	2632185242	97.65	91.88	42.26
HN7	15245796	2256377808	96.96	91.13	41.09	14196146	2079916794	98.05	93.24	41.06
HN8	11048692	1635206416	97.07	91.49	41.30	10274472	1503285246	98.17	93.65	41.27
HN9	8456194	1251516712	96.76	90.96	40.70	7781242	1137831270	97.98	93.31	40.70
HN10	15468192	2281558320	96.82	91.16	41.01	14251198	2076954604	98.02	93.47	41.01
HN11	8496170	1253185075	97.76	93.45	45.18	8069088	1170215374	98.56	95.00	45.11
HN12	7247862	1069059645	96.97	91.18	41.18	6718796	980366949	98.11	93.42	41.16
HN13	10344690	1515497085	97.63	93.11	41.51	9774568	1415306525	98.51	94.83	41.44

HN14	8194050	1200428325	96.96	91.61	42.11	7571376	1095820546	98.14	93.87	42.10
HN15	10331398	1513549807	97.32	92.20	41.63	9691902	1403039985	98.31	94.17	41.58
HN16	10144876	1486224334	96.79	90.98	41.24	9380252	1357286764	97.98	93.27	41.23
HN17	18387170	2684526820	96.72	90.78	40.83	16967902	2447823617	97.93	93.10	40.83
HN18	11672708	1704215368	97.54	92.75	43.82	11042424	1590964119	98.43	94.50	43.74
HN19	10073942	1470795532	97.06	91.90	41.15	9346494	1349033470	98.19	94.05	41.14
HN20	6073968	886799328	96.95	91.27	41.42	5642966	814619521	98.08	93.44	41.40
BH1	13855184	2022856864	97.41	92.52	44.68	12994978	1873831540	98.38	94.41	44.57
BH2	27459840	4009136640	97.21	92.10	41.92	25583004	3691898103	98.22	94.05	41.86
BH3	13352412	1949452152	97.53	92.82	42.37	12587870	1815445045	98.42	94.57	42.31
BH4	18814494	2765730618	97.18	91.98	46.44	17500132	2536476045	98.24	94.02	46.41
BH5	22998732	3380813604	96.97	91.24	42.59	21414580	3112443554	98.07	93.37	42.55
BH6	40875156	6008647932	95.80	87.99	43.04	37348290	5413743609	97.14	90.53	42.99
BH7	30559032	4492177704	96.93	91.32	41.90	28347420	4116966612	98.05	93.47	41.85
BH8	5594706	822421782	97.34	92.18	43.84	5247592	757675045	98.21	93.82	43.71

BH9	19522422	2889318456	96.96	91.41	43.05	18117528	2647131771	98.08	93.56	43.00
BH10	5573442	824869416	97.51	92.82	42.28	5204618	755760834	98.38	94.50	42.05
BH11	28170330	4169208840	97.12	91.76	42.37	26276334	3837894924	98.13	93.70	42.28
BH12	47197212	6961588770	96.92	91.27	42.34	43759416	6374756838	98.02	93.38	42.27
BH13	5705606	841576885	97.02	91.38	42.17	5312830	774585398	98.11	93.51	42.13
BH14	38648892	5700711570	96.78	90.84	45.64	35802876	5210227698	97.92	93.01	45.65
BH15	40967064	6001674876	97.24	92.18	41.93	40965540	5929878195	97.22	92.14	41.84
BH16	5528526	809929059	97.24	92.19	42.06	604968	87102970	97.26	92.20	43.02
BH17	11569988	1695003242	97.40	92.35	40.91	10902320	1579771309	98.34	94.20	40.87
BH18	7544148	1105217682	96.89	91.16	42.53	7008166	1013250482	98.00	93.29	42.49
BH19	10991130	1604704980	97.25	91.89	46.54	10321380	1483909974	98.21	93.77	46.49
BH20	5621740	820774040	97.39	92.30	46.26	5286932	758563179	98.30	94.07	46.17
FCG1	15618448	2280293408	96.72	90.92	41.48	14332094	2067292219	97.97	93.30	41.48
FCG2	7565218	1104521828	96.82	91.15	43.18	6967372	1004664659	98.02	93.45	43.14
FCG3	9528260	1391125960	96.86	91.27	44.21	8772282	1264504498	98.06	93.57	44.19

FCG4	15170978	2214962788	97.19	91.81	42.16	14168630	2045549476	98.23	93.86	42.12
FCG5	6012158	877775068	97.54	92.79	42.23	5664582	817067679	98.43	94.55	42.16
FCG6	20965336	3081904392	96.73	90.96	43.91	19187098	2784987852	97.99	93.36	43.89
FCG7	15483518	2276077146	97.00	91.24	42.36	14391244	2090918854	98.11	93.43	42.33
FCG8	6027980	886113060	96.09	88.86	43.51	5526684	801134096	97.38	91.31	43.48
FCG9	13423442	1973245974	96.56	90.40	41.53	12278168	1782599734	97.85	92.87	41.53
FCG10	11486892	1688573124	96.09	88.96	46.31	10456430	1514193878	97.47	91.57	46.27
FCG11	5461530	808306440	96.65	90.67	42.57	4998530	730184091	97.94	93.14	42.52
FCG12	7700236	1139634928	96.57	90.37	43.42	7044932	1028585685	97.87	92.88	43.41
FCG13	12811990	1896174520	97.15	91.66	43.63	11953682	1746005874	98.20	93.74	43.58
FCG14	14168042	2089786195	96.67	90.66	44.26	13020312	1894189778	97.91	93.04	44.21
FCG15	8701796	1283514910	96.87	90.79	43.26	8069268	1176414956	98.03	93.09	43.22
FCG16	13818430	2038218425	96.63	90.61	42.95	12661514	1842900262	97.91	93.06	42.94
FCG17	6151776	901235184	96.91	91.45	41.68	5663450	819592731	98.11	93.77	41.67
FCG18	6742944	987841296	96.71	90.94	42.80	6165194	891822945	98.00	93.42	42.78

FCG19	30524940	4471903710	97.19	91.78	40.98	28558260	4139771874	98.24	93.85	40.95
FCG20	6162162	902756733	96.91	90.86	41.94	5738754	831661970	98.02	93.03	41.90
ZJ1	6282588	917257848	96.77	90.86	41.49	5813728	838229938	97.95	93.11	41.49
ZJ2	18975876	2770477896	97.38	92.34	40.94	17838182	2575046859	98.35	94.26	40.91
ZJ3	5746022	838919212	97.06	91.86	41.09	5328918	769046249	98.19	94.04	41.09
ZJ4	19401138	2832566148	96.94	91.58	42.71	17897418	2580311367	98.13	93.85	42.67
ZJ5	21006036	3066881256	96.98	91.72	41.00	19409838	2800307625	98.16	93.97	41.02
ZJ6	19917640	2907975440	97.39	92.43	40.97	18703412	2700732100	98.36	94.34	40.94
ZJ7	15493884	2262107064	96.38	89.69	41.96	14194188	2047222642	97.67	92.16	41.94
ZJ8	11808488	1724039248	96.68	90.64	41.16	10867016	1568267722	97.91	93.02	41.16
ZJ9	12943324	1889725304	96.52	90.31	45.61	11797600	1700228328	97.86	92.86	45.57
ZJ10	16488664	2407344944	97.06	91.45	41.56	15390974	2225619602	98.12	93.51	41.54
ZJ11	16470730	2404726580	97.17	91.77	41.56	15413346	2229410599	98.20	93.76	41.53
ZJ12	9810456	1432326576	96.95	91.06	42.25	9127634	1319861815	98.04	93.18	42.21
ZJ13	19614334	2863692764	97.29	92.25	41.04	18354550	2650373463	98.31	94.24	41.02

ZJ14	20998704	3086809488	96.95	91.66	42.30	19333446	2807230038	98.15	93.95	42.26
ZJ15	18373380	2700886860	96.87	91.29	41.02	16985484	2466434361	98.05	93.56	41.03
ZJ16	23441820	3445947540	97.04	91.42	41.41	21936462	3183788163	98.07	93.41	41.36
ZJ17	17850494	2624022618	97.26	92.17	40.35	16665032	2422782935	98.29	94.18	40.31
ZJ18	6512108	957279876	97.03	91.40	42.40	6041016	876709973	98.10	93.46	42.28
ZJ19	20232078	2994347544	97.00	91.30	42.62	18752140	2743062409	98.13	93.51	42.55
ZJ20	21245052	3144267696	96.74	90.90	42.95	19547088	2855650767	97.98	93.27	42.90
FJ1	5424092	802765616	96.81	90.83	46.56	5004412	730821051	97.97	93.04	46.50
FJ2	22027554	3249064215	97.42	92.54	41.18	20632796	3007475319	98.37	94.39	41.08
FJ3	24296562	3583742895	97.06	91.81	43.81	22563060	3281253225	98.17	93.93	43.72
FJ4	15363024	2266046040	96.97	91.38	40.75	14248268	2075786804	98.11	93.59	40.73
FJ5	18668492	2734934078	96.88	91.23	41.29	17265038	2499896962	98.04	93.46	41.25
FJ6	7124148	1043687682	97.56	93.05	40.88	6704932	970808231	98.48	94.84	40.79
FJ7	7210778	1056378977	97.29	92.36	42.01	6760052	977766670	98.29	94.27	41.94
FJ8	6498534	952035231	96.79	90.85	42.67	6033002	872937089	97.92	93.02	42.59

FJ9	5517016	786241096	98.28	94.23	41.73	5401336	766176410	98.69	95.04	41.68
FJ10	10143590	1480964140	96.84	90.83	41.76	9400654	1357916712	98.00	93.10	41.72
FJ11	18487486	2699172956	96.69	90.82	41.91	16936236	2443684847	97.96	93.25	41.88
FJ12	7136620	1041946520	97.10	91.63	41.96	6649396	960059634	98.19	93.75	41.91
FJ13	6051468	883514328	97.21	92.06	42.16	5652078	816109238	98.24	94.06	42.09
FJ14	5737970	837743620	97.14	91.71	42.85	5351020	772443041	98.20	93.78	42.80
FJ15	11203178	1635663988	97.09	91.83	42.65	10423364	1504590645	98.17	93.90	42.60
FJ16	7982602	1173442494	97.64	93.36	44.62	7506612	1086516558	98.51	95.03	44.47
FJ17	18301214	2690278458	96.73	90.79	42.68	16872706	2450434811	97.94	93.10	42.64
FJ18	6886872	1012370184	96.56	89.92	41.73	6393362	928726554	97.72	92.16	41.71
FJ19	7782852	1144079244	97.15	91.66	44.66	7247064	1051603513	98.19	93.71	44.57
FJ20	5761790	846983130	96.52	89.78	44.85	5286922	767310158	97.76	92.20	44.79
Total	14004254.96	2055710963.98	96.99	91.39	42.53	12984224.24	1882838216.86	98.07	93.48	42.50

Table S2 RAD-seq sequencing data and SNP sites statistics

Raw reads	Raw base	Clean reads	Clean base	Q20%	Q30%	GC%	Total SNP	Total SNP after filter
14004255	2055710964	12984224	1882838216	98.07%	93.48%	42.50%	3622965	158264

Table S3 Distribution of SNP sites by sequencing depth range

Depth Range	SNP sites	Percentage (%)	Mean depth
<5X	0	0	
5-10X	62,175	39.28	
10-15X	62,282	39.35	
15-20X	20,258	12.80	15.18X per locus
20-25X	8,543	5.40	
25-30X	3,484	2.20	
>30X	1,522	0.97	

Table S4 Distribution of SNP sites by missing data rate

Missing Rate Range	SNP sites	Percentage (%)	Mean missing rate
0-10%	0	0	
10-20%	633	0.40	
20-30%	22,980	14.52	38.87%
30-40%	49,141	31.05	
40-50%	85,510	54.03	
50-100%	0	0.00	

Table S5 Distribution of SNP sites across heterozygosity ranges

Heterozygosity Range	SNP sites	Percentage (%)	Mean observed heterozygosity
0-10%	32,539	20.56	
10-20%	64,477	40.73	
20-30%	26,161	16.53	20.22%
30-50%	34,454	21.77	
>50%	633	0.40	
